# Persistence of the Gypsy Moth Pheromone, Disparlure, in the Environment in Various Climates

**DOI:** 10.3390/insects4010104

**Published:** 2013-01-14

**Authors:** Ksenia S. Onufrieva, Kevin W. Thorpe, Andrea D. Hickman, Donna S. Leonard, E. Anderson Roberts, Patrick C. Tobin

**Affiliations:** 1 Department of Entomology, Virginia Tech, Blacksburg, VA 24061, USA; E-Mails: anhickma@vt.edu (A.H.); roberts@vt.edu (E.R.); 2 49 Hart Road Spencer, NY 14883, USA; E-Mail: kthorpe11@gmail.com; 3 USDA Forest Service, Forest Health Protection, Asheville, NC 28802, USA; E-Mail: dleonard@fs.fed.us; 4 USDA Forest Service, Northern Research Station, Morgantown, WV 26505, USA; E-Mail: ptobin@fs.fed.us

**Keywords:** Lymantria dispar, pheromone, mating disruption, environmental persistence

## Abstract

Mating disruption techniques are used in pest control for many species of insects, yet little is known regarding the environmental persistence of these pheromones following their application and if persistence is affected by climatic conditions. We first studied the persistent effect of ground applications of Luretape® GM in *Lymantria dispar* (L) mating disruption in VA, USA in 2006. The removal of Luretape® GM indicated that the strong persistent effect of disparlure in the environment reported by previous studies is produced by residual pheromone in the dispensers as opposed to environmental contamination. In 2010 and 2011, we evaluated the efficacy of two formulations, Disrupt® II and SPLAT GM^TM^, in VA and WI, USA, which presented different climatic conditions. In plots treated in WI and VA, male moth catches in pheromone-baited traps were reduced in the year of treatment and one year after the pheromone applications relative to untreated controls. However, similar first- and second-year effects of pheromone treatments in VA and WI suggest that the release rate over one and two years was the same across markedly different climates. Future applications that use liquid or biodegradable formulations of synthetic pheromones could reduce the amount of persistence in the environment.

## 1. Introduction

Techniques that disrupt successful mating are used as a management tactic against a number of insect pests, especially against Lepidoptera [1,2,3,4]. The method of mating disruption is based upon the introduction of synthetic pheromone into the environment at levels that chemically interfere with mate-finding ability. Compared to insecticides, mating disruption has the advantage of having no known adverse effects to non-target species, and even the target species is not killed. The precise mechanism of mating disruption remains unknown, although it is believed that several mechanisms might be at work simultaneously: competitive attraction, false trail following, camouflage of the plume produced by calling females by the airborne artificial pheromone, desensitization, habituation, and sensory imbalance [5,6]. For mating disruption to be successful as an insect management tactic, synthetic pheromone needs to be present in the air in sufficient quantities over the course of the mating period [7,8,9]. Previous studies have highlighted that synthetic pheromones, when deployed as part of insect pest management programs, could persist in the environment beyond the year of application, producing a ‘ghost effect’ [10,11,12]. The mechanisms of pheromone persistence in the environment are not fully understood. It has been shown that pheromone can be adsorbed onto foliage under field conditions [13]. Therefore, persistence of some synthetic pheromones could result from environmental contamination in which bark, foliage, and leaf litter adsorb and re-emit pheromone over time [10,11,14]. For example, the gypsy moth, *Lymantria dispar* (L.) (Lepidoptera: Lymantriidae), sex pheromone (disparlure, cis-7,8-Epoxy-2-methyloctadecane) is a relatively large molecule (MW = 282.504 g/mol) and is characterized by a large partitioning coefficient, which allows it to adsorb onto solid surface and to evaporate later, thus potentially producing a significant “ghost effect” [13].

In the case of aerial mating disruption applications, residual pheromones could also be due to pheromone dispensers that remain on the ground and continue to release pheromone beyond the year of application. Dispensers with a synthetic form of disparlure that were aerially applied as a mating disruptant were shown to contain 1.8–23.2% of their initial disparlure one year after application [15]. Previous studies have also shown that disparlure, when introduced through aerial application of controlled release dispensers, persists in the environment, resulting in significantly reduced female mating success and male moth trap catch up to 2 years following application [12].

Mating disruption is the primary treatment tactic against *L. dispar* under the USDA Forest Service-Cooperating State Slow-the-Spread (STS) program [16]. The goal of the STS program is to reduce the rate of expansion of *L. dispar* in the USA by detection and elimination of low-density isolated colonies that are located just beyond the expanding population front of the infested area [16,17]. *Lymantria dispar* was introduced to North America and continues to expand its range [18]. Established populations occasionally erupt to outbreak densities that tend to be spatially synchronized, resulting in widespread economic and ecological damage over a relatively short time period [19,20]. Larvae are polyphagous folivores that can exploit over 300 deciduous and coniferous host trees [21]. 

Approximately 1,200–1,600 km^2^ are treated each year in the STS program using mating disruption tactics. Previous studies have reported that aerially applied synthetic disparlure persists beyond the year of treatment [12,15], although the mechanism remains unclear. Regardless, this persistent effect poses advantages and disadvantages. For example, the STS program relies upon a sophisticated internet-based data management system and decision algorithm to manage the volume of collected data and assist in decision making [16]. Specifically, male moth density data from a grid of pheromone-baited traps are used to identify newly-formed isolated colonies and treat them before they grow and coalesce with the population front. Therefore, despite the potential advantages of the continued reduction of mating success of females after mating disruption applications, the continued suppression of male moth trap catch also impairs the evaluation of treatment effectiveness and potentially leads to an underestimation of the residual population density in treated areas. An underestimation of *L. dispar* population density or the failure to detect a new population due to the residual effects of pheromones from previous mating disruption applications has important management implications; for example, such populations may not be detected until later when they are greater in density, more spatially widespread, and consequently more expensive to manage [22,23]. 

The objectives of the study were two-fold. First, we sought to test if short- and long-term persistence of synthetic disparlure following application was due to pheromone dispensing agents left in the field or environmental contamination. Second, we sought to determine the persistent effect of synthetic disparlure when applied in two climatically different environments, VA and WI, which represent two climate extremes within the current area where the STS program is implemented. We were motivated by the importance of climate because past work conducted under both laboratory and field conditions have shown that the rate of disparlure release is temperature-dependent [24,25,26,27,28,29].

## 2. Materials and Methods

### 2.1. Mechanism of Short- and Long-term Persistence

Two blocks, each consisting of three plots (190 × 190 m in size), were selected in the Little North Mountain Wildlife Management Area, Rockbridge County, VA, USA (38.0631° N, 79.3244° W to 38.0596° N, 79.3315° W). In each block, 1 plot was used to monitor short-term persistence (over the course of 1 week), 1 plot was used to monitor long-term persistence (over the course of a summer field season), and 1 plot was left untreated and used as a control for both treatments. The distance between treated plots was ≥1 km. The experimental plots were treated with Hercon Luretape® GM (Hercon Environmental, Emigsville, PA). Similar to the aerially applied formulation of disparlure, Hercon Disrupt® II (Hercon Environmental, Emigsville, PA), Luretape® GM is a three-layered plastic laminated dispenser. It is 3.8 cm wide, has 2 polyvinyl chloride (PVC) outer layers, and contains racemic disparlure at a concentration of 12.9 mg/cm^2^ [30]. This controlled-release formulation is a standard for use in ground applications against L. dispar populations.

**Figure 1:**
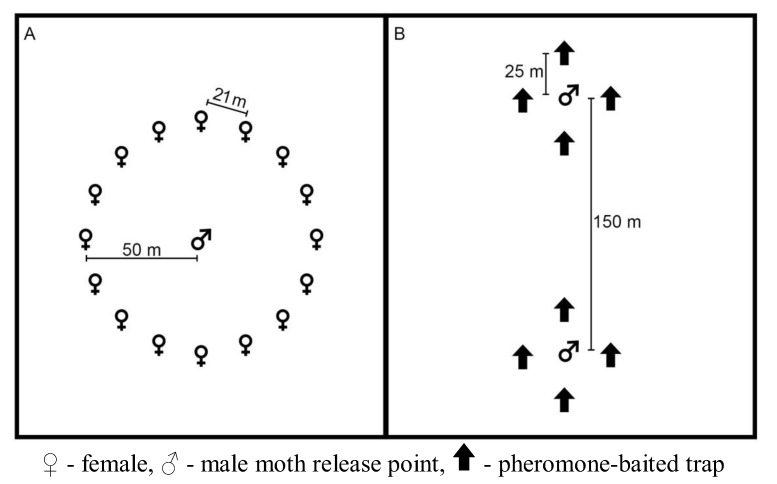
Generalized plot layout for the short- and long-term pheromone persistence (**A**) and pheromone persistence between two climate regimes (**B**) experiments.

In our short-term plots, we applied Luretape® GM at a rate of 75 g active ingredient (AI)/ha and at a density of 40 point sources per ha. Luretape® GM was applied by tying 90 cm long strips of the dispenser to tree branches ≈2 m from the ground on June 21, 2006. We evaluated the effect of the pheromone on *L. dispar* mating success by deploying tethered females. Fifteen laboratory-reared virgin females (≤24 hours old) were placed on tree boles 1.5 m from the ground and protected from predation by a band of duct tape covered with Tanglefoot bird repellent (The Tanglefoot Company, Grand Rapids, MI, USA). Females were deployed for 24 hours four times a week in a 50-m radius circle around adult male release points, which were located at the centers of the treated and control plots (Figure 1A) [31]. Collected females were maintained under laboratory conditions for at least 60 days to allow for embryonization. Egg masses were individually inspected to ascertain successful mating [32]. Laboratory-reared males (≈150) were released twice a week at the time of female deployment. We monitored the experimental and control plots for 3 consecutive iterations. An iteration consisted of 2 weeks: one week with the Luretape® GM present in the experimental plots followed by a week with the Luretape® GM removed from the plots. 

In our long-term plots, we applied Luretape® GM at the same rate as our short-term plots. In these plots, Luretape® GM was placed in the field on June 15, 2006 and left in place until August 8, 2006. Plots were monitored for 8 weeks beginning on June 19, 2007 by alternating the deployment of tethered females and pheromone-baited traps, which were USDA milk-carton pheromone traps baited with 500 μg of (+)-disparlure in twine dispensers [25]. Tethered females and pheromone-baited traps were never deployed at the same time due to potential interference between sources of pheromone [33]. Each study plot had a male moth release point established at the center of the plot. We deployed 15 females (≤ 24 hours old) in the same manner as our short-term plots. Four pheromone-baited traps were placed 25 m beyond the circle of deployed females. For each week of the 8 week study period, ≈100 males were released daily. Females were deployed on Mondays and Wednesdays, and on Tuesdays and Thursdays females were removed and traps were deployed.

We used logistic regression (PROC GENMOD, SAS Institute 2008) to analyze mating success of females in control and treated plots. Deployed females were scored as either mated or not, and this binary variable was adjusted by the number of females returned (as despite our efforts, some females still succumbed to predation) and modeled as a function of treatment, block, iteration, and block by treatment interaction. Significance was based on the likelihood ratio chi-squared, G^2^, and when appropriate, we estimated odds ratios and associated confidence intervals. We used ANOVA with Tukey’s adjustment for multiple comparisons (PROC GLM, SAS Institute 2008) to analyze male moth catches from pheromone-baited traps. The total moth counts per trap per week were transformed using ln (N+1) and were modeled as a function of treatment and block. The interaction of treatment and block was used as an error term.

### 2.2. Pheromone Persistence Between Two Climate Regimes

**Figure 2:**
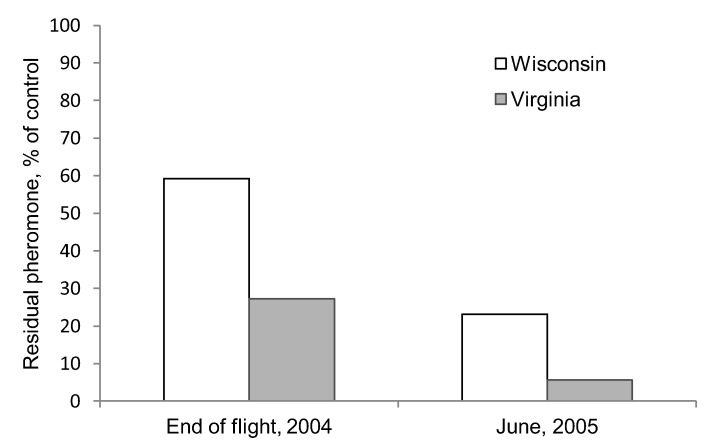
Residual amount of pheromone left in Hercon Disrupt® II plastic flakes after 10 weeks of exposure during the summer flight period and in the following year in VA and WI. The residual amounts of disparlure are shown as percentages based on control dispensers.

Prior to the field studies, we first conducted a preliminary experiment in 2004 at one site in Bayfield County in northern WI, USA (46.5358° N, 91.1953° W) and at one site in the Appomattox-Buckingham State Forest, Appomattox County, VA (37.54167°N, 78.45889°W) to quantify residual pheromone concentration at the end of male moth flight period and at the beginning of the next year. We glued 20 Hercon Disrupt® II flakes impregnated with disparlure to 125 10 × 20 cm canvas coated paper cards (Strathmore Paper Co., Westfield, MA) using a multipolymer emulsion glue that is used as a sticking agent in aerial applications of Disrupt® II (Gelva 2333, Solutia Inc., Springfield, MA, USA). Twenty five of these cards were placed immediately in a freezer (–10 °C) for use as a control. At each site, 50 cards were hung on lines under the canopy, approximately 1.5 m from the ground, at the beginning of each site’s male moth flight period. After 10 weeks, 25 cards were collected and placed in a freezer. Following leaf drop, the remaining cards were then covered with litter and placed into window screen cages and left on the ground over the winter until the following April. The amount of residual pheromone was determined using a Model 6C-9A gas chromatograph (Shimadzu Instruments, Columbia, MD) [34]. We observed that at the end of the flight season, roughly twice as much pheromone was left in the flakes deployed in WI than those deployed in VA. Following the winter, the flakes deployed in WI still had ≈4 times more pheromone than those deployed in VA (Figure 2). Because these results indicated a slower release rate at our colder site, we hypothesized that the efficacy of mating disruption treatments, which depend upon a sufficient quantity of pheromone being released to disrupt mating communication, could be affected by climate. Consequently, we designed a study to directly compare first- and second-year effects of mating disruption treatments in VA and Northern WI. 

In 2010–2011, we evaluated the persistence of synthetic disparlure in the Goshen Wildlife Management Area, VA, USA (38.0631 °N, 79.3244 °W to 38.0596 °N, 79.3315 °W) and in the Northern Highland American Legion State Forest, WI, USA (46.1123 °N, 89.4296 °W to 45.9379 °N, 89.6703 °W) using two formulations of disparlure, Hercon Disrupt® II and SPLAT GM^TM^ (ISCA Tech, Riverside, CA, USA). The mean January and July temperatures in the Goshen Wildlife Management Area are 1 and 24 °C, respectively. In the Northern Highland American Legion State Forest, the respective mean temperatures are –12 and 19 °C; furthermore, in this State Forest, mean temperatures are <0 °C from November to March (National Climatic Data Center 2012). Thus, these two study areas provided different climate conditions, and also were representative of the southern and northern extent of the operational area of the STS program. 

In 2010, at each of our two study areas, we selected six plots, each 500 × 500 m in size and separated by ≥1 km. The plots were grouped into 2 blocks with 3 plots per block. In each block at the WI site, we had one untreated control plot, one plot treated with Disrupt® II at 15 g AI/ha, and one plot treated with SPLAT GM^TM^ at 15 g AI/ha. In each block at the VA site, we had one untreated control plot, one plot treated with Disrupt® II at 15 g AI/ha, and one plot treated with SPLAT GM^TM^ at 11.4 g AI/ha due to an application error. Because SPLAT GM^TM^ was not applied correctly, we did not monitor plots in VA for second-year effect. Instead we used the results of a study conducted in the Goshen Wildlife Management Area VA in 2008 using the same protocol. Six 500 × 500 m plots separated by ≥1 km were selected and grouped into 2 blocks with 3 plots per block. In the first block, one plot was treated with Hercon Disrupt® II at 15 g AI/ha, one plot was treated with SPLAT GM^TM^ at 15 g AI/acre and one plot was left untreated and used as control. In the second block, SPLAT GM^TM^ was applied at 11.9 g AI/acre due to an application error; the other treatments were the same as in the first block. The plots were treated in 2007 and evaluated in 2008. 

The Disrupt® II formulation consisted of plastic flakes composed of PVC outer layers and an inner polymer layer containing 17.9% racemic disparlure. The flakes were mixed with diatomaceous earth (3% wt/wt) to reduce clogging and were aerially applied using a fixed-wing aircraft (Air Tractor) equipped with specialized application pods (Schweitzer Aircraft Corp., Elmira, NY, USA). Within the pods, the flakes were mixed with Gelva 2333 and dispensed through a spinner [9]. Disparlure release rate from applied flakes was not determined in this study. However, in previous studies where plastic flakes were applied under similar conditions, the flakes released 30–50% of their disparlure content over the 6-week period of male moth flight [15,34].

SPLAT GM^TM^ is a liquid formulation that is designed for both aerial and ground application. The formulation contains 13.0% racemic disparlure and is applied as mixture dollops of various sizes using conventional application systems pressurized either by positive displacement pumps, pressurized gas cylinders or a combination of both. SPLAT GM^TM^ was applied using a Beechcraft King Air fixed-wing aircraft. Disparlure release rate from applied microcapsules was not determined in this study. A Global Positioning Satellite (GPS) navigation system was used to guide all spray applications [35].

The efficacy of each treatment in disrupting *L. dispar* mating was evaluated by the recapture of released laboratory-reared males. Each study plot had 2 male moth release points located 150 m to the north and south of the plot center (Figure 1B). Each male moth release point was surrounded by 4 standard USDA milk-carton pheromone traps baited with 500 μg of (+)-disparlure in twine dispensers (Hercon Environmental Corporation, Emigsville, PA, USA), placed 25 m to the north, south, east and west of the release point. Male gypsy moths were obtained as pupae from the USDA Animal and Plant Health Inspection Service, Pest Survey Detection and Exclusion Laboratory, OTIS Air National Guard Base, MA, USA. Pupae were kept in laminated paper cups with Saran wrap lids. We released ≈100 adult males at each release point twice a week. The pheromone-baited traps were checked and emptied at the time of each release. 

We used a General Linear Model ANOVA procedure with Tukey’s adjustment to test for significance of differences in moth counts between groups of traps located in plots treated with various formulations of pheromone for each of the studies. To analyze trap catch in the year of pheromone application, the log-transformed total moth counts per trap per week for each type of pheromone treatment, ln(*N*+1), was modeled as a function of state, week, pheromone treatment, and block with interactions of factors. For the analysis of the pheromone effect one year after the application, the log-transformed total moth counts per trap per week for each type of pheromone treatment, ln(*N*+1), was modeled as a function of pheromone treatment, block and week with interactions of factors. The pheromone treatment by block interaction was used as an error term. To ensure comparable male moth density among plots and to extend the time period during which the data could be collected, we considered only laboratory-reared males in our statistical analyses. Laboratory-reared males were distinguished from background male moths by a dye that was added to the larval diet and subsequently visible at the base of the wings. 

## 3. Results

### 3.1. Mechanism of Short- and Long-term Persistence

**Figure 3:**
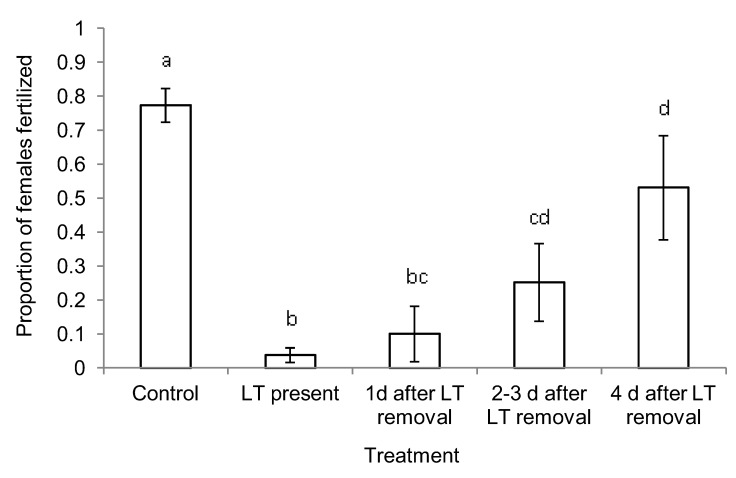
Proportion of females fertilized (± SE) in plots treated with Luretape® GM (LT) in Goshen Wildlife Management Area, VA in 2006. Bars with the same letter are not significantly different.

In the short-term exposure experiment, mating success of females was significantly reduced by treatments compared with mating success in the untreated control plots (G^2^ = 168; df = 63; *P *< 0.01). In the presence of Luretape® GM, female mating success was reduced to 4.8% of that in control plots, and individual females were estimated to be 292 times (95% CI = 53, >999) less likely to be mated when deployed in treated plots compared to control plots. The removal of Luretape® GM resulted in a gradual increase in mating success that ranged from 13% after 1 day to 69% after 4 days (Figure 3). One day after the removal of Luretape® GM, female mating success did not differ from plots where Luretape® GM was still present (G^2^ = 1.08; df=1, 21; *P *= 0.3); yet, females from plots where Luretape® GM had been removed for one day were still 100 times (95% CI = 18, 580) less likely to be mated than females from untreated control plots. Four days after Luretape® GM removal, females were 13 times (95% CI = 2, 94) more likely to be mated than in plots treated with Luretape® GM, but 8 times (95% CI = 3, 23) less likely to be mated than females in untreated control plots. In the long-term exposure experiment, in which treatment was applied in 2006 and data evaluated in 2007, both the mating success of females (G^2 ^= 0.14; df = 35; *P *= 0.7) and male moth catch from pheromone-baited traps (F = 0.5; df= 1, 24; P = 0.6) were not significantly different between treated and untreated control plots.

### 3.2. Pheromone Persistence between Two Climate Regimes

**Figure 4:**
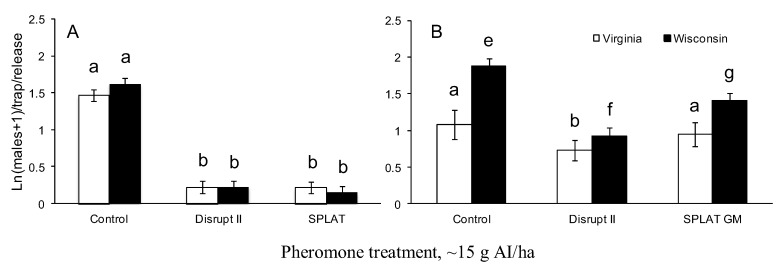
Male moth trap catch from pheromone-baited traps in the year of pheromone treatment (**A**) and one year after the pheromone treatment (**B**). Within (A), bars with the same letters are not significantly different, within (B), for each region (VA or WI), bars with the same letters are not significantly different.

In both VA and WI, male moth trap catch was significantly suppressed by all pheromone treatments relative to untreated control plots in the year of treatment (F = 211; df = 2, 159; P < 0.0001; Figure 4A). Trap catches in treated plots relative to untreated control plots were suppressed to >94% and >95% in Virginia and WI, respectively. There was no significant interaction between study area and treatment (F = 0.22; df = 2, 159; P = 0.8), indicating that the treatments were equally effective in VA and WI in the year of treatment.

One year after treatment application, trap catches in treated plots in WI were still significantly suppressed by all treatments compared to control plots (F = 20; df = 2, 39; P < 0.0001; Figure 4B). The trap catches in plots treated with Disrupt® II and SPLAT GM^TM^ were suppressed by 70% and 44%, respectively, compared to untreated control plots. In VA, the same applications of Hercon Disrupt®II reduced trap catches by 53% compared to untreated control plots (F = 20; d.f. = 2, 40; P = 0.0003, Figure 4B), while SPLAT GM^TM^ reduced trap catches by 29% compared to control plots. The differences between trap catches in plots treated with SPLAT GM^TM^ and untreated controls in VA were not significant (Figure 4B). 

## 4. Discussion

The gradual increase in mating success following the removal of Luretape® GM (Figure 3) suggests that disparlure may have been adsorbed by components of the environment (e.g., bark, foliage, and litter) and re-emitted, resulting in the short-term mating disruption. This finding is in agreement with a previous study that quantified the evaporation rate of pheromones [13]. The persistent short-term reduction in mating success relative to untreated control plots and after the removal of the pheromone sources could have also been facilitated by the fairly large size of the disparlure molecule. It is also possible that dense foliage around our plots promoted pheromone build up by reducing wind speed and consequent pheromone dilution [36].

However, the adsorption and re-emission of pheromone following the removal of the sources did not persist in the year following treatment, as neither mating success nor trap catch differed between treated and untreated control. A previous study documented the persistent effect of aerial applications of disparlure up to two years following application [12]. Our observations of the increase in mating success following disparlure removal in combination with the lack of long-term persistent effect of disparlure suggests that the second-year effects previously observed [12] resulted from continued emissions of pheromone from plastic laminated dispensers beyond the year of application as opposed to long-term environmental contamination. We do note, however, that the results provide evidence of the possibility of short-term environmental contamination.

When considering the persistence of disparlure between two different climate conditions, the analysis of the residual amount of disparlure in Disrupt® II plastic flakes indicated that the same pheromone treatments produce less airborne pheromone in WI than in VA, suggesting that the same treatments may become less effective in the year of application and produce stronger persistent effect in WI compared to VA. However, the level of male moth trap catch suppression was similarly observed in WI and VA in the year of application. One year after the application, the level of mating disruption in plots treated with Disrupt® II was similar to a previous study conducted in VA, where Disrupt® II applied at the same rate as in our studies reduced trap catch by 50–80% compared to untreated control plots [12]. In both states, one year after the application, the liquid formulation SPLAT GM^TM^ produces a weaker second-year effect than the plastic flake formulation Hercon Disrupt® II. Since SPLAT GM^TM^ is a liquid formulation, it is applied as a mixture of dollops of different sizes. The smaller dollops degrade faster than the larger ones and the number of point sources is constantly reduced. This may explain the difference in the second-year effects of aerial SPLAT GM^TM^ applications between VA and WI. In VA, SPLAT GM^TM^ may be degrading faster than in WI due to higher temperatures and increased exposure to UV radiation. Both of these factors are known to affect mating disruption [13].

We observed similar effects of disparlure, whether formulated as Hercon Disrupt® II plastic flakes or liquid SPLAT GM^TM^, and whether treatments were conducted in a warmer (VA) or cooler (WI) climates. In the year of application, both formulations appear to release pheromone at adequate rates over a range of temperatures, which allows using them in all of the U.S. The long-term residual effect of disparlure following treatment does have advantages, because it extends the temporal efficacy of the application. However, in certain cases, the multi-year persistence of pheromone following aerial application could be problematic, such as when it obfuscates subsequent trap catch data. This could be particularly problematic in eradication programs that rely on a rapid assessment of population density. In such cases, it could be more prudent to use liquid or biodegradable formulations that may evaporate or biodegrade more quickly than those involving plastic laminated dispensers.

In many insect pest management strategies, the persistence of a control tactic is generally considered to be advantageous if it reduces the need for multiple applications, thereby reducing overall management costs. However, long-term persistence of insect control tactics is rarely desired, especially for chemical insecticides that can affect a broader range of taxa. Although some degree of persistence is desirable for mating disruption techniques, persistence from generation-to-generation, or in the case of *L. dispar* from year-to-year, could complicate the interpretation of data used to evaluate treatment efficacy. In some cases, it could even lead to a false conclusion that the target population has been sufficiently reduced or eliminated. Understanding the causes of persistence and quantifying the rate of persistence from year-to-year following the deployment of synthetic pheromones under mating disruption tactics enhances the overall utility of this control tactic.

## References

[1] Carde R.T., Minks A.K. (1995). Control of moth pests by mating disruption: successes and constraints. Annu. Rev. Entomol..

[2] El-Sayed A.M., Suckling D.M., Wearing C.H., Byers J.A. (2006). Potential of mass trapping for long-term pest management and eradication of invasive species. J. Econ. Entomol..

[3] Suckling D.M., Tobin P.C., McCullough D.G., Herms D.A. (2012). Combining tactics to exploit Allee effects for eradication of alien insect populations. J. Econ. Entomol..

[4] El-Sayed A.M., Mitchell V.J., Manning L.-A.M., Suckling D.M. (2011). New sex pheromone blend for the lightbrown apple moth, *Epiphyas postvittana*. J. Chem. Ecol..

[5] Miller J.R., Gut L.J., de Lame F.M., Stelinski L.L. (2006). Differentiation of competitive *vs.* non-competitive mechanisms mediating disruption of moth sexual communication by point sources of sex pheromone (Part 2): Case studies. J. Chem. Ecol..

[6] Yamanaka T. (2007). Mating disruption or mass trapping? Numerical simulation analysis of a control strategy for lepidopteran pests. Popul. Ecol..

[7] Onufrieva K.S., Brewster C.C., Thorpe K.W., Sharov A.A., Leonard D.S., Reardon R.C., Mastro V.C., Sellers P., Roberts E.A. (2008). Effects of the 3M (TM) MEC sprayable pheromone (R) formulation on gypsy moth mating success. J. Appl. Entomol..

[8] Wins-Purdy A.H., Judd G.J.R., Evenden M.L. (2008). Mechanisms of pheromone communication disruption in choristoneura rosaceana exposed to microencapsulated (Z)-11-tetradecenyl acetate formulated with and without horticultural oil. J. Chem. Ecol..

[9] Thorpe K., Reardon R., Tcheslavskaia K., Leonard D., Mastro V. (2006). A review of the use of mating disruption to manage gypsy moth, *Lymantria dispar (L.)*; FHTET-2006–13.

[10] Karg G., Suckling D.M., Bradley S.J. (1994). Absorption and release of pheromone of *Epiphyas postvittana* (Lepidoptera: Tortricidae) by apple leaves. J. Chem. Ecol..

[11] Suckling D.M., Karg G., Bradley S.J. (1996). Apple foliage enhances mating disruption of light-brown apple moth. J. Chem. Ecol..

[12] Thorpe K.W., Tcheslavskaia K.S., Tobin P.C., Blackbum L.M., Leonard D.S., Roberts E.A. (2007). Persistent effects of aerial applications of disparlure on gypsy moth: Trap catch and mating success. Entomol. Exp. Appl..

[13] Gut L.J., Stelinski L.L., Thomson D.R., Miller J.R. (2004). Behaviour-modifying Chemicals: Prospects and Constraints in IPM.

[14] Wall C., Sturgeon D.M., Greenway A.R., Perry J.N. (1981). Contamination of vegetation with synthetic sex-attractant released from traps for the pea moth, Cydia nigricana. Entomol. Exp. Appl..

[15] Leonhardt B.A., Mastro V.C., Leonard D.S., McLane W., Reardon R.C., Thorpe K.W. (1996). Control of low-density gypsy moth (Lepidoptera:Lymantriidae) populations by mating disruption with pheromone. J. Cheml. Ecol..

[16] Tobin P.C., Blackburn L.M. (2007). Slow the Spread: A National Program to Manage the Gypsy Moth.

[17] Sharov A.A., Leonard D., Liebhold A.M., Roberts E.A., Dickerson W. (2002). Slow the Spread": A national program to contain the gypsy moth. J. Forest..

[18] Tobin P.C., Bai B.B., Eggen D.A., Leonard D.S. (2012). The ecology, geopolitics, and economics of managing the gypsy moth, *Lymantria dispar* (L.), in the United States. Int. J. Pest Manage..

[19] Johnson D.M., Liebhold A.M., Bjornstad O.N., McManus M.L. (2005). Circumpolar variation in periodicity and synchrony among gypsy moth populations. J. Anim. Ecol..

[20] Haynes K.J., Liebhold A.M., Johnson D.M. (2009). Spatial analysis of harmonic oscillation of gypsy moth outbreak intensity. Oecologia.

[21] Elkinton J.S., Liebhold A.M. (1990). Population-Dynamics of Gypsy-Moth in North-America. Ann. Rev. Entomol..

[22] Epanchin-Niell R.S., Haight R.G., Berec L., Kean J.M., Liebhold A.M. (2012). Optimal surveillance and eradication of invasive species in heterogeneous landscapes. Ecol. Lett..

[23] Liebhold A.M., Tobin P.C. (2006). Growth of newly established alien populations: comparison of North American gypsy moth colonies with invasion theory. Popul. Ecol..

[24] Nation J.L., Foltz J.L., Dixon W.N., McAuslane H.J. (1993). Evaluation of loss of (+)-disparlure from gypsy moth (Lepidoptera: Lymantriidae) pheromone dispenser tapes under field conditions in Florida. Fla. Entomol..

[25] Leonhardt B.A., Mastro V.C., Devilbiss E.D. (1992). Evaluation of pheromone dispensers for use in gypsy moth detection (Lepidoptera: Lymantriidae). J. Entomol. Sci..

[26] Leonhardt B.A., Mastro V.C., Paszek E.C., Schwalbe C.P., Devilbiss A.D. (1990). Dependence of gypsy moth (Lepidoptera: Lymantriidae) capture on pheromone release rate from laminate and other dispensers. J. Econ. Entomol..

[27] Bierl-Leonhardt B.A., DeVilbiss E.D., Plimmer J.R. (1979). Rate of release of disparlure from laminated plastic dispensers. J. Econ. Entomol..

[28] Leonhardt B.A., Moreno D.S., Leonhardt B.A., Beroza M. (1982). Evaluation of controlled release laminate dispensers for pheromones of several insect species. Insect Pheromone Technology: Chemistry and Applications.

[29] Tobin P.C., Zhang A., Onufrieva K., Leonard D.S. (2011). Field evaluation of effect of temperature on release of disparlure from a pheromone-baited trapping system used to monitor gypsy moth (Lepidoptera: Lymantriidae). J. Econ. Entomol..

[30] Kolodny-Hirsch D.M., Webb R.E., Olsen R., Venables L. (1990). Mating disruption of gypsy moth (Lepidoptera: Lymantriidae) following repeated ground application of racemic disparlure. J. Econ. Entomol..

[31] Thorpe K.W., Hickman A.D., Tcheslavskaia K.S., Leonard D.S., Roberts E.A. (2007). Comparison of methods for deploying female gypsy moths to evaluate mating disruption treatments. Agr. Forest. Entomol..

[32] Tcheslavskaia K., Brewster C.C., Sharov A.A. (2002). Mating success of gypsy moth (Lepidoptera : Lymantriidae) females in Southern Wisconsin. Great Lakes Entomol..

[33] Elkinton J.S., Carde R.T. (1988). Effects of Intertrap Distance and Wind Direction on the Interaction of Gypsy-Moth (Lepidoptera, Lymantriidae) Pheromone-Baited Traps. Environ. Entomol..

[34] Thorpe K.W., Mastro V.C., Leonard D.S., Leonhardt B.A., McLane W., Reardon R.C., Talley S.E. (1999). Comparative efficacy of two controlled-release gypsy moth mating disruption formulations. Entomol. Exp. App..

[35] Onufrieva K.S., Thorpe K.W., Hickman A.D., Tobin P.C., Leonard D.S., Roberts E.A. (2010). Effects of SPLAT® GM sprayable pheromone formulation on gypsy moth mating success. Entomol. Exp. App..

[36] Karg G., Sauer A.E., Koch U.T. The Influence of Plants on the Development of Pheromone Atmospheres Measured by EAG Method. Proceedings of 18th Gottingen Neurobiology Conference.

